# Amoral, im/moral and dis/loyal: Children’s moral status in child welfare

**DOI:** 10.1177/0907568217711742

**Published:** 2017-06-08

**Authors:** Zlatana Knezevic

**Affiliations:** Mälardalen University, Sweden

**Keywords:** Agency, child welfare, assessment framework, discourse, feminist theory

## Abstract

This article is a discursive examination of children’s status as knowledgeable moral agents within the Swedish child welfare system and in the widely used assessment framework BBIC. Departing from Fricker’s concept of epistemic injustice, three discursive positions of children’s moral status are identified: amoral, im/moral and dis/loyal. The findings show the undoubtedly moral child as largely missing and children’s agency as diminished, deviant or rendered ambiguous. Epistemic injustice applies particularly to disadvantaged children with difficult experiences who run the risk of being othered, or positioned as reproducing or accommodating to the very same social problems they may be victimised by.

Making moral and knowledge-based judgements is a common practice in the social services, not least within the child welfare system that is often assigned the moralising task of discerning the ‘good’ from the ‘evil’. There are many accounts of how children and childhood figure as the very object of moralising practices and moral valuation, and as a means to justify various statements and actions. This is the case also in child welfare moral orders. While children and childhood have a symbolic link to both morality and knowledge acquisition (Mayall, 2000; Meyer, 2007; Parton, 2014), little is known about actual children’s opportunities to embody the position of the knowledgeable moral subject in, what otherwise seems to be, knowledge-oriented child welfare policy and practice.

The article aims at exploring child service users’ status as knowledgeable moral subjects by examining how children are positioned discursively in the context of the Swedish child protection policy and by turning to *BBIC*, the widely used framework for assessment in Sweden. BBIC (abbreviation for ‘Children’s Needs in Focus’ [sw. *Barns Behov i Centrum*]) was introduced for national implementation by the Swedish National Board of Health and Welfare in 2006. Children’s participation and influence is one of the aims of the framework, which mirrors recent developments towards child-centred and participation-oriented child welfare (Gilbert et al., 2011; NBHW, 2015a: 8, 17). Given that the policy is targeting vulnerable children at risk of harm, the analysis focuses particularly on some of the possible implications of the available positions regarding vulnerable children and children’s experiences of social problems.

The concept of epistemic injustice serves as a theoretical point of departure for this article (Fricker, 2007). Extensive contributions from feminist and postcolonial theory show that epistemic injustice is tightly interlinked with moral status and that the knowing subject implies a certain degree of trustworthiness in order for her knowledge to be seen as valid and reliable (Alcoff, 1996; Jaggar, 2000; Spivak, 1999). By raising questions about epistemic injustice, the article argues that a child- and participation-oriented child welfare is not sufficient when tackling children as service users. A central argument is that if children are to participate in the context of child welfare investigations and assessments so they can have a say and opportunity to influence decisions about their lives, it is imperative to grant them recognition as knowledgeable agents who are capable of moral reasoning. By casting light on moral status as shaped by ageist, classed, gendered and racialised constructions, the article provides a more multifaceted description of children facing epistemic injustice as service users. As will be argued below, this is a particular challenge when considering socially disadvantaged children and children in vulnerable situations facing social problems within the family.

## Epistemic injustice and moral status of children

This article draws upon critical childhood researchers’ re-readings of feminist and postcolonial theory and the long-running debate surrounding the construction of the knowing subject. Conceptually, Miranda Fricker’s (2007) *epistemic injustice* is used as an umbrella term. It denotes a systematic deficit in trustworthiness and denial of someone’s capacity as a knower due to structural relations of power, that is, class, ethnicity/‘race’, gender and sexuality as well as age. In the many accounts of how knowledge is intertwined with power, it becomes manifested how those depicted as less knowledgeable are also depicted as morally inferior or unreliable (Alcoff, 1996; Fricker, 2007; Murris, 2013; Spivak, 1999). What has been discussed as epistemic trust, credibility or trustworthiness is in this article referred to as *moral status*.^1^ The concept is used as a delineation in relation to the umbrella term epistemic injustice, hence analytically separated. Moral status entails primarily two meanings: the capacity to make moral judgements and the status granted these judgements. This may address the entitlement to make knowledge and moral claims and have the privilege of interpretation. It also addresses the uneven distribution of trustworthiness due to structural power relations (Alcoff, 2010; Fricker, 2007; Murris, 2013).

According to feminist and postcolonial theory, the degrading depiction of women as irrational and the colonial Others’ preconceived infantilism is at once a prerequisite for and an invention derived from the Western conceptualisation of the subject, traditionally ascribed to the Enlightened white European male adult (Alcoff, 1996; Jaggar, 2000; Spivak, 1999). Critical childhood researchers have used this in analogy to the developing child, showing that many of those attributes accorded to childhood and children – incompetence, immaturity, irrationality as well as unreliability and moral underdevelopment and deviance – are in direct reversal of this conventional conceptualisation of the subject (Burman, 2017; Mayall, 2000; Nieuwenhuys, 2013; Robinson, 2012; Sundhall, 2012). While in postcolonial theory this refers to the presumed supremacy of whiteness, justifying the civilising missions directed at the uneducated colonial Others (Brown, 2005; Burman, 2017), in critical childhood studies this corresponds to the idea that adults ought to educate children in moral standards. As a result, the moral authority and responsibility of adults simultaneously exclude the possibility to think about children as moral agents (Mayall, 2000). Critical contestations of this depiction of children as passive objects of parental influence instead portray children as social agents who can make a difference to the social world (James, 2011).

The still prevailing ideas about childhood as the formative stage of the adult-to-be put the developing passive child at the core of developmental psychology, socialisation theory and social anthropology, to name a few (Alanen, 1988; James et al., 1998). It has been argued that similar to the image of the pre-historic colonial Others that have been used to depict the Western trajectory to civilisation, the pre-subjectial child is supposed to reveal something about cultural reproduction and socialisation (Burman, 2017; Castañeda, 2001; James et al., 1998; Sundhall, 2012). These ideas have also been influential when studying the causes, outcomes and transmission of social problems and risk (Kaufman and Zigler, 1989; Vinnerljung, 1998). While the ideas date far back to the late 19th century and the child study movement’s incorporation of evolutionary ideas (Burman, 2017), in Sweden, these ideas commonly refer to the influential ‘founding father’ of Swedish child welfare work, the child psychiatrist Gustav Jonsson in the 1960s, and his theory about the inheritance of social problems across generations. His theory pictures ‘delinquent boys’ in care as products of biological parents’ and grandparents’ problems and puts them at the centre when dealing with the problematic Others of social work and child welfare (Jonsson, 1967, 1973). Child welfare, being grounded in both protection and surveillance, reflects the ‘moral panics’ of its time, entailing anxieties about negative impacts on children, constructing children as both potentially innocent victims and monstrous Others (Parton, 2014; Thorne, 1987). When constructed as shaped by parents but without moral agency on their own, children’s moral competence and credibility undergoes constant questioning and children, as a group, are often projected with a ‘dubious moral status’ (Mayall, 2000: 257).

The line of argument resonates with Fricker’s *hermeneutical injustice*, meaning that certain experiences of children’s agency and children’s moral reasoning cannot be articulated within ‘the collective hermeneutical resources’ (Fricker, 2007: 155) about children and childhood. One such hermeneutical injustice, as Eriksson (2009) shows, is the challenge for the child service user to simultaneously embody the position of a victim *and* a competent agent. This may especially be the case when a child’s social position is not in alignment with the cultural aged and gendered ideas about a passive incompetent feminised victimisation (Eriksson, 2009). Equally, when agency is recognised, children’s low epistemic and moral status tend to portray this agency as negative and problematic, especially when children do not conform to the moral and social orders of professionals assessing them or when children belong to the less privileged groups in society (Franck and Nilsen, 2015; Iversen, 2014; Murris, 2013). Moral status, hence, is unevenly distributed, and along the lines of class, ethnicity/‘race’, gender and sexuality as well as age (Fricker, 2007) also when it comes to individuals belonging to the social group of children (Burman, 2017; Murris, 2013; Robinson, 2012).

A more detailed discussion about possible similarities and differences between adults and children as epistemic and moral agents goes beyond the scope of this article. However, it should be noted that when it comes to the analysis presented below, it departs from the presumption that children are able to recognise right from wrong and what is socially acceptable or not, and that they are able to make moral assessments and judgements in relation to their own and others’ actions.

## Material and methodology

In Sweden, the assessment of children at risk is undertaken by the municipality child welfare services. In this article, I look at child welfare policy by drawing on the guiding framework for such assessments, BBIC, as an empirical example. BBIC is currently used by 285 (280 licenced) out of 290 municipalities (NBHW, 2016). The full version of BBIC was first issued by the Swedish National Board of Health and Welfare in 2006 as a primer inspired by the British Integrated Children’s System and adapted to Swedish legislation (for an overview, see Matscheck and Eklundh, 2015). A second version came in 2013, and a third, more thorough, revision was published in 2015. The aim of launching BBIC was similar to that in England and Wales, that is, to aid systematisation to assessments, to provide equal services throughout the country and to strengthen the position of the child service user (Matscheck and Eklundh, 2015; NBHW, 2006, 2013, 2015a). The conceptual framework for BBIC is an adaptation from the British system and is linking primarily to the developmental ecological perspective. It also includes ‘other theories about children’s and young people’s development’, with attachment theory and developmental psychopathology explicitly stated (NBHW, 2006: 20-21, 2013: 26-28). Modelled by these theoretical influences are also the three main areas – child development, parenting capacity, and family and environment – which together constitute ‘the BBIC triangle’ and provide guidance in assessments.

While the analysis focuses on the more recent primer from 2015 (NBHW, 2015a), it is also informed by readings of the earlier versions (NBHW, 2006, 2013). Another document included in the material, *Assessing children’s maturity for participation* (NBHW, 2015b), is chosen as it addresses child service users’ participation and their epistemic status more in depth. The findings below are a spin-off from an analysis of the system’s prevailing discourses on social problems and risk as they emerge in the developmental conceptual framework and the underpinning theoretical influences in BBIC. Such influences identified were the theory of social heredity, socialisation theory and attachment theory as well as influences from psychopathology and criminology, which together made visible contradictory *discursive positions* of vulnerable child service users at risk of harm. As new questions started to emerge, the focus of the analysis shifted to the positioning of children in the texts under study (Foucault, 1982; Hall, 2001), paying attention to epistemic and moral issues in the contexts of social problems and risk. Apart from the empirical material highlighting social problems and risk, passages addressing children’s participation, knowledge and moral status were added to the analysis.

Methodologically, the article is inspired by the Foucauldian tradition of analysis of discourse (Foucault, 1974, 1982, 1991). Discourse, if defined as ‘a particular way talking about and understanding the world (or an aspect of the world)’ (Jørgensen and Phillips, 2002: 1), sets limits to approaches to the world by creating boundaries between the unthinkable and the taken for granted. This applies to available locations, discursive positions in a discursive terrain that determines the ways in which individuals are made into subjects who ‘personify the discourse’ (Foucault, 1982; Hall, 2001: 73). The analytical strategy was to search for statements about social problems and risk, after which discursive positionings of children – derived through recurrent themes, overlaps, discontinuities, absences and contradictions – were identified (Carabine, 2001; Foucault, 1991; Hall, 2001; Jørgensen and Phillips, 2002). Three overarching positions were discerned and interpreted as distinctively epistemic. Given their relatedness to disparate moral claims and uneven distribution of trustworthiness, the positions identified have social implications for child service users’ participation status. Naming these as the positions of the *amoral child*, the *im*/*moral child* and the *dis*/*loyal* child mirrors the interpretative process as well as points to the concept of epistemic injustice that particularly applies to moral issues. The disposition of the analysis is organised in accordance with this logic as well as the associated discourses identified. Below, I discuss this particularly with regard to children’s capacity to form and express moral judgements and the moral status of these judgements.

## The amoral child

Children’s capacity to form and express moral judgements is, for instance, touched upon in the BBIC primer under the subheading ‘creating opportunities for participation’ when children’s attitudes towards social services are discussed, and where a passive position particularly applying to younger children emerges:

Whether the contact for a younger child becomes a positive experience depends to a large extent on if the parents perceive the social services as comprehensible, meaningful and somewhat predictable. (NBHW, 2015a: 17, author’s translation)

The quote’s focus on parents’ views on the social services mirrors the emphasis put in the BBIC framework on transparency, trust and good collaboration between social services, parents and the child (NBHW, 2015a: 17). This focus, though, implies the portrayal of a child whose views on the social services are reduced to her parents’ views. The text’s positioning of children as *amoral* denotes this lacking capacity to forming independent moral judgement.

The amoral child position is ‘familialized’ (Alanen, 1988) as it reinforces the idea that children are shaped especially by (biological or adoptive) parents and not by any adult figures, that is, social workers, to the same extent. Familialisation is, furthermore, depicted in the sense that the family, rather than the individuals that are part of it, is portrayed as holding certain thoughts about state institutions. This may be interpreted as implicitly linking to the *discourse of social heredity* and the ‘antisocial family syndrome’ which depicts some families as holding negative thoughts of discontent about state institutions, displaying suspicion and treatment resistance (Jonsson, 1967: 15). If seen in this light, the amoral child’s incapability of forming autonomous judgement and her capability for taking in judgements of the parents make the amoral child and the problematic anti-social family mutually constitutive constructs.

The discourse of social heredity, however, becomes more accentuated in relation to social problems and risk, and ‘the intergenerational hypothesis’ (Kaufman and Zigler, 1989: 129). In BBIC, this appears under the heading ‘Family background’:

Risk for the child: To have parents who have been victimised when growing up

An adult person’s capacity to be a parent can be influenced by his or her own experiences from growing up. A risk factor for inadequate parental capacity and indirect risk for the child can thus be if the parent has been growing up with violence, substance misuse, mental health problems or other serious adversities in the family. (NBHW, 2015a: 28, author’s translation)

Here, social problems link back to parents’ upbringing and implicitly also to child service users’ childhood experiences as determining what future generation will be at risk of experiencing. In this discursive formation (Foucault, 1974) on social heredity of problems and risks as transmitted across generations, an attachment discourse^2^ linking to early childhood as well as a discourse on socialisation are mutually supportive of the construction of childhood. The child is positioned as a passive object for parents’ influence in a uni-linear parent-to-child transmission. From the point of moral status, moral conducts of previous generations shape childhood rather than the child herself. When depicted in this way, childhood resembles a formative stage and a site of ‘human ontology’ that reveals something about social and cultural reproduction or socialisation of a future citizen (Castañeda, 2001; James, 2011; James et al., 1998; Mayall, 2000; Robinson, 2012). The quote’s tendency to focus on childhoods, rather than children themselves, supports the idea of tracking backwards to a family of origin, (presumably) biological parents. A passage about unaccompanied refugee children and the early stages of childhood can illustrate this point:

It can be hard to see the point of assessing the parents’ capacities when the child no longer is in their care. However, it is important to understand that even if the child’s parents are still in the country of origin or at some other place in the world they still influence the child in different ways. For example, the child’s previous experiences of the parents’ care, stimulation and guidance or the occurrence of violence may influence how the child can confide in and trust other adults. If the child is missing and worry a lot about siblings, parents and friends it can impact upon the child’s physical and mental health. All of this may be important information when creating support interventions for the child. (NBHW, 2015a: 78, author’s translation)

What I want to highlight here is that the unaccompanied child’s weaker, if at all existing, links to the parents do not make the parenting capacity, now part of a pastime, any less important for assessing the impact on the child and, for instance, her presumed dis/trust of ‘other adults’. It means that potential trust issues and suspicion may link to children themselves and their early experiences, rather than actual trustworthiness of others in the present. A child’s own moral assessment of a adult subjects’ trustworthiness, whether parents’ or social workers’, seem to be beyond the capabilities available for the amoral child.

Although BBIC advocates children’s participation, the position of the amoral child reflects a logic where children’s voice is reduced to that of parents and where children’s participation and versions of things could, in practice, be substituted with parents’. The uni-linear parent-to-child transmission discussed above raises questions about children’s presumed capability of assessing their parents’ and other adults’ actions in moral terms. Facing a problem at home such as gender-based violence, parents’ substance misuse or any other problem that constitutes risk according to BBIC would in this view not imply the possibility for the child to take a moral and judgmental stance against it. Instead, the quotes above produce the position of the amoral child and of children’s agency that is of the reproductive rather than the resisting kind, especially when living in a family with social problems.

## The im/moral child

There are also discourses which do produce children as able to make independent judgements. Nonetheless, such agency tends to be depicted as morally deviant, hence the positioning of children as *immoral*. The passage below can serve as an example, collected from the part of the primer discussing child development, and an identity based on ‘rebellious, deviant, anti-social or criminal values’. Another term mentioned is ‘defiant’:

Risk associated with the child: to display rebellious, anti-social, criminal values and attitudes

To have an identity based upon rebellious, deviant, anti-social or criminal values and attitudes constitute risk factors for anti-social, criminal behaviour, but also for long-term psychosocial problems. (NBHW, 2015a: 45, author’s translation)

Being defiant can constitute a risk for the child. It is therefore important to pay attention to the child defying the wishes of parents’ and others’ and their reprimands, or easily becomes very angry and irritated. (NBHW, 2015a: 46, author’s translation)

In these accounts, children are, unlike the amoral passive child, depicted as either refusing to accept the transmitters’ ‘wishes’ or capable of taking a stance that goes against the wishes of parents and other adults. A *discourse of juvenile delinquency*, underpinning this position, portrays young service users as threats to the moral order of the adult culture (Brown, 2005; Thorne, 1987). In such a discourse, there is no room for unjust or ‘defiant’ adults, nor legitimate resistance towards them (Iversen, 2014; e.g. Nieuwenhuys, 2013). Instead, children’s resistance in itself denotes immorality and risk (Brown, 2005; Franck and Nilsen, 2015), indicating that critical assessments of parents are beyond the scope of this child position. My analysis shows that when positioned in this way, children may be talking without being heard within a collective hermeneutical repertoire in which they ‘need not be understood, but must simply be controlled’ (Brown, 2005; Smith, 2009: 253).

It can be noted that these accounts seem to be applicable primarily to children in their adolescent years. With the shift from the discourse of social heredity to the discourse of juvenile delinquency, there is also a shift in conception of risk. From risk being linked to parents with younger children, in adolescence risk links to children themselves. However, once risk is downplayed and participation is foregrounded, adolescent youth are depicted as competent and with increased moral abilities, as stated under the heading ‘The capacity to tell’ in the document *Assessing children’s maturity for participation*:

At the age of 11-14 the child becomes more able to draw logically grounded conclusions and to follow the logics of verbal accounts. The older teenagers can in a more nuanced way reason about conflicts between moral rules, social conventions and personal choices. They also get a more and more extensive experience of making decisions independently, but may have difficulties in stopping quick decisions that ‘feel good’ in the situation. (NBHW, 2015b: 18, author’s translation)

In the quote, a differentiation is made between different ages, suggesting that older children (11–14 years of age) possess an increased capacity for logic and ‘reason about conflicts between moral rules, social conventions and personal choices’. At the same time, children are constructed as emotionally rather than rationally driven, and as showing impulsiveness and thus lack of forethought and consideration of long-term consequences.

When prior experiences are added to the picture however, this positioning of children’s maturity also becomes open for modification. In the same document, it is stated:

However, every individual follows their own developmental course. For instance, maturity may be affected by crises; a child who has been through tough decisions previously (perhaps due to a chronic illness or care neglect) may show greater maturity in some respects than their peers – or conversely, may be delayed in their maturity development. (NBHW, 2015b: 15, author’s translation)

Research shows that information, experience, environment, social and cultural expectations and the extent of support helps in developing a child’s ability to form opinions. There are many indications that children’s previous experiences to a large extent form the basis for competence and maturity of the child, rather than age and stage of development. (NBHW, 2015b: 16, author’s translation)

The quotes put vulnerability at the heart of maturity assessments. Individualisation of maturity on the basis of prior experiences, such as hardships and crises, is in focus, rather than age and developmental level. Problems in childhood are thereby seen as influencing maturity development, and consequently children’s moral status, which opens up for an ambiguous, *im*/*moral*, positioning. These children may be seen as both more mature and less mature than their peers when it comes to forming opinions. While not necessarily to their disadvantage in the assessment, it still suggests that children previously exposed to vulnerability should be assessed differently, hence othered. As shown, the immoral child position may be underpinned by a discourse of juvenile delinquency and enhance the vulnerability of children who do not live up to cultural expectations of what a child should be or how they should behave (Brown, 2005; e.g. Robinson, 2012). The passages in the quote above about children’s maturity add difficult childhood experiences to the equation. Yet dissimilar to the case of the amoral child, the quotes are not about inheriting or adopting parents’ judgements. The focus is instead on the hardships themselves. The targeted and othered children are still the same, those living with or having experienced difficult circumstances that set them apart and make them more likely to face epistemic injustice.

## The dis/loyal child

In the material, a third position can be found, which draws on prior discursive positions but grants children an ambiguous moral status in relation to loyalty issues. For example, under the heading of ‘violence, abuse and exploitation’, the primer draws attention to children not talking about the violence at home:

Violence, abuse and exploitation are often family secrets that children do not tell anybody else about. This can be due to children’s feelings of fear and confusion, loyalty, guilt or shame. Therefore there is a risk that their vulnerability is not recognised or only partly recognised. (NBHW, 2015a: 51, author’s translation)

When interpreted as a moral virtue, loyalty, together with ‘feelings’ of guilt, shame, confusion and fear, presupposes moral capacity in children. However, in the passage above, loyalty may also stand in the way of knowledge, that is, telling others and giving a correct and informative testimony about family secrets.^3^ Victims’ silence about violence could be interpreted as linked to power and oppression (Enander and Holmberg, 2008), but in this case the primer seems to draw on the *discourse of child loyalty.* Supporting this interpretation are the statements in the quote about children being loyal towards their parents, and children being silent due to their loyalty, or statements about loyalty conflicts and, thus, conflicts between moral imperatives (Christianson et al., 2013; Solberg, 2007: 32). Such taken-for-granted assumptions about children’s loyalty discourage the surrounding adults from asking about and hearing children’s versions of things (Christianson et al., 2013; Solberg, 2007). In contexts of gender-based violence, researchers show how children choose their loyalties by taking stance against violence and how they may even encourage their mothers to leave their violent partners (Katz, 2015; Solberg, 2007). In BBIC, however, children are loyal to parents while secretive and disloyal as service users – hence ambiguously positioned as *dis*/*loyal*.

In contrast to the im/moral child, the loyal child is ascribed traits of obedience, silence and faithfulness and more generally emotionality that conventionally is ascribed the feminised private sphere of family (Jaggar, 2000). When foregrounding loyalty towards parents rather than social workers, children’s deliberative epistemic ‘loyalties’ towards the subjective (rather than the objective, public, and common good) and the emotional (as opposed to the rational, i.e. professionals’ distanced approach) are also highlighted (Alcoff, 1996; e.g. Mayall, 2000; NBHW, 2015a: 14). Here, it is possible to link to previous discussions of representations of children as naive (Meyer, 2007; Robinson, 2012), a naivety that becomes accentuated when children show loyalty towards their family members at home, and implicitly also their problematic norms and behaviours. Thus, from an epistemic point of view, loyalty based on emotions is an irrational loyalty. This may render the dis/loyal child service user unreliable even in the epistemically low position as provider of ‘raw information’, a position from which she is not even granted ‘the rational capacity of interpretation and judgement that is generally accorded to subjects’ (Alcoff, 2010: 131; Fricker, 2007).

While the discourse of child loyalty primarily links to child–parent relationships, in cross-cultural families, it may also denote the loyalty conflicts between multiple cultures:

Those children and young people growing up with several cultures (both in families with foreign background and in those that belong to a national minority) often form a cross-cultural identity, with elements from both parents’ culture of origin as from the surrounding majority society. It is an asset and provides a special competence, linguistically and socially alike. However, it may also lead to conflicting loyalties. This may be the case if they feel that their parents have a difficulty accepting the ‘Swedish’ part of their identity, or if the society is discriminating against their foreign or minority background […] (NBHW, 2013: 46, author’s translation)

Here, the discourse of child loyalty merges with a national discourse on loyalty as ethno-cultural belonging (e.g. Schmitt, 2010), portraying a child standing between the majority culture and the culture of the parents. While granted particular competences, a child with a cross-cultural upbringing may also experience conflicting loyalties. Thus, the quote’s tendency to depict issues of power, that is, discrimination, as issues of loyalty, can also be found when it comes to this child position. The impression is one of a conflicting loyalty or ‘dubious moral status’ (Mayall, 2000: 257) in the child *herself* as a result of the presumed value conflicts in her surroundings. The depiction of a presumed disloyalty as a result of discrimination positions children as potentially unreliable, albeit agentic. Such a positioning is not seldom linked to the Others that are already disadvantaged socially (Burman, 2017; Fricker, 2007; Murris, 2013).

A similar positioning also appears when a child’s dis/loyalty towards parents is depicted. Unlike the child that is aligning herself with the parents due to oppression in the family, here, intolerance within the family becomes a reason *not* to align. Having explored similar presumptions in the context of the United States, Ong (2003) shows how adolescent girl service users from minority cultures may be granted greater agency in decision-making than their peers. Yet, this is rooted in a gendered and racialised construction of victimisation where young girls ought to be rescued from patriarchal and traditional parental Others. While all parties are disadvantaged due to institutional racism and cultural othering, the status of children may differ from that of their parents in how it is shaped by age, ethnicity/‘race’ and gender (Ong, 2003). Thus, the depiction of children belonging to ‘several cultures’ may open up for a more agentic and independent, nonetheless, culturally essentialised position. Unlike the positioning of the amoral child, the dis/loyal child’s moral status is not entirely reduced to the parents, that is, familialised. Nevertheless, children belonging to minority cultures are still linked to their (biological) parents through a reductionism in relation to culture (Brown, 2005; Ong, 2003). The inconsistent positioning of majority and minority children within the discourse of child loyalty produces the risk of epistemic injustice through differentiation of children’s moral status on the grounds of ethnicity/‘race’.

## Discussion

This article discusses child service users’ status as knowledgeable moral agents in an era when knowledge has gained a lot of attention in social work, not least in developments towards evidence-based practice and a more knowledge-oriented child welfare system. However, as shown above, the child service user’s knowledgeability is contested, undermined or minimised, indicating epistemic injustice at play. While not making any claims to a comprehensive covering of possible discursive positionings of children’s moral status within Swedish child welfare policy and practice, my analysis gives particular attention to three positions that make this form of injustice visible, and this concluding section is further discussing the implications for service users’ participation in investigation, assessment and decision-making within the child welfare system.

While there is a variation in moral status among the position of the amoral child, the im/moral child and the dis/loyal child, none of them positions children as undoubtedly moral agents. Apart from constructing children as reproducers of the same attitudes that their parents may hold, the position of the amoral child is linked to a presumption that children as people are not capable of assessing their parents’ actions in moral terms. If seen in this way, children victimised by their parents’ wrongdoings may be mistrusted and problematised in two different ways. First, it becomes quite futile for children to participate as their presumed inability to give an independent testimony is what precisely marks them as amoral. Second, they run the risk of being seen as potentially problematic themselves, especially considering the future outcomes of their previous victimisation. The interventions produced by such a positioning therefore become measures where the child is ought to be saved from parents’ wrongdoings in order not to become like them, while – paradoxically – simultaneously reduced to them and familialised. This impossibility to be regarded autonomously and in their own right, or to express morally justified resistance towards the parents becomes especially problematic in situations where social problems remain invisible for others than the child. Making problems at home visible for case workers requires the child to identify or disclose a problem. However, this requires moral judgements and distinguishing good from bad, thus a capacity for moral reasoning which children in all three positions are constructed as predominately lacking.

Furthermore, epistemic injustice as an analytical tool helps illuminate that children’s tenuous moral position and the idea that children are unable of moral reasoning should not be entirely linked to a discussion about cognitive capacity or children’s age. With increased age, children are not necessarily presumed to display equally increased moral capacity. The position of the im/moral child makes this particularly obvious. This positioning makes it easy for adolescents with difficult experiences to be ‘heard’ as deviant, disobedient or impulsive rather than as displaying justified resistance for the sake of their own and others’ best interests. The im/moral child is an agentic position, yet agency is predominantly described as negative or rendered ambiguous. The analysis suggests that children’s morally justified resistance falls outside of the discourses underpinning the child welfare policy framework. It also suggests that children with difficult experiences are othered and set apart in maturity assessments, which makes vulnerable children more likely to face epistemic injustice.

The discourse on child loyalty is drawing on two contrasting notions of the child: the loyalty of the naive attached child and the somewhat morally dubious child. This may depict circumstances of family secrets and silences and construct loyalty towards parents as standing in the way of children providing a correct and disclosing testimony about their circumstances as well as their best interests. Children in cross-cultural families are, in contrast, positioned as more agentic in the face of oppressive circumstances. This autonomy, nevertheless, is still marked as a loyalty issue. Furthermore, the contradictory way of positioning majority and minority children, respectively, creates risk for epistemic injustice on the grounds of ethnicity/’race’

All three positions, the familialised and depersonalised amoral child, the im/moral child and the dis/loyal child, can be linked to previous theorisations about the inequalities that actual children may face as included in the broader category of children. As Fricker and others show, moral status is unevenly distributed and along the lines of age, class, ethnicity/‘race’, gender and sexuality. This article adds vulnerable children living in families with social problems to the picture. The analysis of the discursive positions reveals BBIC as shaped by both paternalism and an essentialism marked primarily by class and ethnicity/‘race’, and as a policy measure with vulnerable children occupying a central role in its ‘moral anxieties’.

A critical analysis of the discourses of social heredity, juvenile delinquency and child loyalty, together with the discursive positions identified, discerns what so far seems to be missing in the BBIC documents: the undoubtedly moral child. The ‘moral child’ is the social agent whose possible resistance and beliefs point to social change rather than to a link in a destructive chain of social problems transferred from one generation to another. This position would be the child who is capable of resisting surroundings that are destructive or unjust for her, when it also implies opposing parents or social services. It is the child who embodies another kind of society, where social problems are explained in ways not necessarily directly linking to the family and parents but also include broader societal issues and structural power relations. This would also allow children’s talk or possible silence about what is going on at home to be interpreted within a context of societal power relations rather than one of loyalty, individual relations or victim blame. In a similar way, resistance towards oppression would not be depicted as a display of distrust, defiance or loyalty conflict, but one of social action in the face of power relations. However, in order for this position to exist, recognition of children’s moral agency is a prerequisite, as well as depicting children as at least partly autonomous, less familialised and more linked to the society as a whole. By pathologising, problematising or othering children’s agency, that is, ascribing it to the anti-social, defiant and/or culturally Others, the moral status of children as such never gets increased, and children’s knowledgeability and capacity for moral reasoning and resistance remain exception rather than a norm.

## Notes

1. Moral status resembles Fricker’s epistemic trust, which is implied in and is part of epistemic injustice (Fricker, 2007). However, here I use moral status to include the contexts of children and childhood where the capability of making moral judgements also needs to be considered as epistemic injustice issue, rather than (merely) a cognitive capability.

2. The focus on early childhood socialisation can be interpreted as grounded in developmental psychology and attachment theory, which puts the emphasis on the two first years during which bonding takes place (Bowlby, 1969).

3. Within the research on gender-based violence, there is a discussion, beyond the scope of this article, about shame or fear as preventing the victim from making the experience of violence known to others. The point I want to make here is that this is rarely depicted as an issue of loyalty but one of power relations (for an overview, see Enander and Holmberg, 2008).

## References

[bibr1-0907568217711742] Alanen L (1988) Rethinking childhood. Acta Sociologica 31(1): 53–67.

[bibr2-0907568217711742] Alcoff LM (1996) Is the feminist critique of reason rational. Philosophic Exchange 26(1): 59–79.

[bibr3-0907568217711742] Alcoff LM (2010) Epistemic identities. Episteme 7(2): 128–137.

[bibr4-0907568217711742] Bowlby J (1969) Attachment and Loss, vol. 1 New York: Basic Books.

[bibr5-0907568217711742] Brown S (2005) Understanding Youth and Crime – Listening to Youth? 2nd edn. Berkshire: Open University Press.

[bibr6-0907568217711742] Burman E (2017) Deconstructing Developmental Psychology, 3rd edn. London; New York: Routledge.

[bibr7-0907568217711742] Carabine J (2001) Unmarried motherhood 1830-1990: A genealogical analysis. In: Wetherell M Taylor S Yates SJ (eds) Discourse as Data: A Guide for Analysis. Los Angeles, CA: SAGE, pp. 267–310.

[bibr8-0907568217711742] Castañeda C (2001) The child as a feminist figuration: Toward a politics of privilege. Feminist Theory 2(29): 29–53.

[bibr9-0907568217711742] Christianson SÅ Azad A Leander L et al (2013) Children as witnesses to homicidal violence: What they remember and report. Psychiatry, Psychology and Law 20(3): 366–383.

[bibr10-0907568217711742] Enander V Holmberg C (2008) Why does she leave? The leaving processes of battered women. Health Care for Women International 29: 200–226. 18350425 10.1080/07399330801913802

[bibr11-0907568217711742] Eriksson M (2009) Girls and boys as victims: Social workers’ approaches to children exposed to violence. Child Abuse Review 18(6): 428–445.

[bibr12-0907568217711742] Foucault M (1974) The Archaeology of Knowledge. London: Tavistock Publications.

[bibr13-0907568217711742] Foucault M (1982) The subject and power. Critical Inquiry 8(4): 777–795.

[bibr14-0907568217711742] Foucault M (1991) Discipline and Punish: The Birth of the Prison. London: Penguin.

[bibr15-0907568217711742] Franck K Nilsen RD (2015) The (in) competent child: Subject positions of deviance in Norwegian day-care centres. Contemporary Issues in Early Childhood 16(3): 230–240.

[bibr16-0907568217711742] Fricker M (2007) Epistemic Injustice: Power and the Ethics of Knowing. Oxford: Oxford University Press.

[bibr17-0907568217711742] Gilbert N Parton N Skivenes M (2011) Changing patterns of response and emerging orientations. In: Gilbert N Parton N Skivenes M (eds) Child Protection Systems. Oxford: Oxford University Press, pp. 243–257.

[bibr18-0907568217711742] Hall S (2001) Foucault: Power, knowledge and discourse. In: Wetherell M Taylor S Yates SJ (eds) Discourse Theory and Practice. London: SAGE, pp. 72–81.

[bibr19-0907568217711742] Iversen C (2014) Predetermined participation: Social workers evaluating children’s agency in domestic violence interventions. Childhood 21(2): 274–289.

[bibr20-0907568217711742] Jaggar AM (2000) Feminist ethics. In: LaFolette H (ed.) The Blackwell Guide to Ethical Theory. Malden, MA; Oxford; Carlton, VIC, Australia: Blackwell Publishing, pp. 348–374.

[bibr21-0907568217711742] James A (2011) Agency. In: Qvortrup J Corsaro WA Honig MS (eds) The Palgrave Handbook of Childhood Studies. New York: Palgrave Macmillan, pp. 34–45.

[bibr22-0907568217711742] James A Jenkins C Prout A (1998) Theorizing Childhood. Cambridge, Oxford: Polity Press.

[bibr23-0907568217711742] Jonsson G (1967) Delinquent boys: Their parents and grandparents. Acta Psychiatrica Scandinavica: Supplementum 195: 1–264. 5230974

[bibr24-0907568217711742] Jonsson G (1973) Att bryta det sociala arvet [Breaking the social heredity]. Stockholm: Folksam/Tidens förlag.

[bibr25-0907568217711742] Jørgensen M Phillips L (2002) Discourse Analysis as Theory and Method. London; Thousand Oaks, CA; New Delhi, India: SAGE.

[bibr26-0907568217711742] Katz E (2015) Domestic violence, children’s agency and mother–child relationships: Towards a more advanced model. Children & Society 29(1): 69–79.

[bibr27-0907568217711742] Kaufman J Zigler E (1989) The intergenerational transmission of child abuse. In: Ciccheti D Carlson V (eds) Child Maltreatment. Cambridge: Cambridge University Press, pp. 129–150.

[bibr28-0907568217711742] Matscheck D Eklundh LB (2015) Does BBIC make a difference? Structured assessment of child protection and support. Nordic Social Work Research 5(3): 193–211.

[bibr29-0907568217711742] Mayall B (2000) The sociology of childhood in relation to children’s rights. International Journal of Children’s Rights 8: 243–259.

[bibr30-0907568217711742] Meyer A (2007) The moral rhetoric of childhood. Childhood 14(1): 85–104.

[bibr31-0907568217711742] Murris K (2013) The Epistemic challenge of hearing child’s voice. Studies in Philosophy and Education 32: 245–259.

[bibr32-0907568217711742] NBHW (2006) Grundbok Barns behov i centrum (BBIC) [Children’s needs in focus (BBIC)]. Stockholm: Socialstyrelsen.

[bibr33-0907568217711742] NBHW (2013) Barns behov i centrum – Grundbok BBIC [Children’s needs in focus]. Stockholm: Socialstyrelsen.

[bibr34-0907568217711742] NBHW (2015a) Barns behov i centrum – Grundbok BBIC [Children’s needs in focus]. Stockholm: Socialstyrelsen.

[bibr35-0907568217711742] NBHW (2015b) Bedöma barns mognad för delaktighet [Assessing children’s maturity for participation]. Stockholm: Socialstyrelsen.

[bibr36-0907568217711742] NBHW (2016) BBIC-kommuner [BBIC municipalities]. Available at: http://www.socialstyrelsen.se/barnochfamilj/bbic/Sidor/bbic-kommuner.aspx#anchor_0 (accessed 17 January 2016).

[bibr37-0907568217711742] Nieuwenhuys O (2013) Theorizing childhood(s): Why we need postcolonial perspectives. Childhood 20(1): 3–8.

[bibr38-0907568217711742] Ong A (2003) Buddha Is Hiding: Refugees, Citizenship, the New America. Berkeley, CA; Los Angeles, CA; London: University of California Press.

[bibr39-0907568217711742] Parton N (2014) The politics of child protection. Contemporary Developments and Future Directions. Basingstoke: Palgrave Macmillan.

[bibr40-0907568217711742] Robinson KH (2012) Childhood as a ‘queer time and space’: Alternative imaginings of normative markers of gendered lives. In: Robinson KH Davies C (eds) Rethinking Research and Professional Practices in Terms of Relationality, Subjectivity and Power. Sharjah, United Arab Emirates: Bentham eBooks, pp. 100–139.

[bibr41-0907568217711742] Schmitt I (2010) ‘Normally I should belong to the others’: Young people’s gendered transcultural competences in creating belonging in Germany and Canada. Childhood 17(2): 163–180.

[bibr42-0907568217711742] Smith R (2009) Childhood, agency and youth justice. Children & Society, 23(4): 252-264.

[bibr43-0907568217711742] Solberg A (2007) Hur förhåller sig barn till våld i hemmet? [Children negotiating domestic violence]. In: Eriksson M (ed.) Barn som upplever våld [Children exposed to violence]. Stockholm: Gothia Förlag, pp. 25–37.

[bibr44-0907568217711742] Spivak GC (1999) A Critique of Postcolonial Reason. Cambridge, MA; London: Harvard University Press.

[bibr45-0907568217711742] Sundhall J (2012) Kan barn tala? En genusvetenskaplig undersökning av ålder i familjerättsliga utredningstexter [Can children speak? A gender studies exploration of age in family law social work reports]. PhD Thesis, Gothenburg University, Sweden.

[bibr46-0907568217711742] Thorne B (1987) Re-visioning women and social change: Where are the children? Gender and Society 1(1): 85–109.

[bibr47-0907568217711742] Vinnerljung B (1998) Socialt arv [Social inheritence]. In: Denvall V Jacobson T (eds) Vardagsbegrepp i socialt arbete [Frequent concepts in social work]. Stockholm: Norstedts Juridik, pp. 21-44.

